# Reply to Herbrecht et al

**DOI:** 10.1093/cid/ciaa208

**Published:** 2020-03-02

**Authors:** John W Baddley, C Orla Morrissey, Cornelia Shaefer-Prokop, Sharon C-A Chen, Peter G Pappas, J Peter Donnelly

**Affiliations:** 1 University of Maryland School of Medicine, Baltimore, Maryland, USA; 2 Alfred Health and Monash University, Melbourne, Australia; 3 Meander Medical Center Amersfoort and Radiology, Radboud University Medical Center, Nijmegen, The Netherlands; 4 Centre for Infectious Diseases and Microbiology, Laboratory Services, Institute of Clinical Pathology and Medical Research, Westmead Hospital, University of Sydney, Sydney, Australia; 5 Department of Medicine, Division of Infectious Diseases, University of Alabama at Birmingham, Birmingham, Alabama, USA; 6 Department of Hematology, Radboud University Medical Center, Nijmegen, The Netherlands

To the Editor—We thank Herbrecht and colleagues for their valuable comments regarding application of the updated radiologic criteria of the revised and updated consensus European Organization for Research and Treatment of Cancer and the Mycoses Study Group Education and Research Consortium (EORTC/MSGERC) definitions for invasive fungal disease [1]. The authors point out that an important limitation of the first definitions in 2002 [2] and the 2008 iteration [3] was an overly strict definition of radiologic signs of pulmonary disease. In the most recent version of the definitions, the finding of a wedge-shaped, lobar or segmental consolidation was added to the existing criteria of dense, well-circumscribed lesion(s) with or without a halo sign, air crescent sign, or cavity, which, together with a host criterion and mycologic evidence, constitute probable invasive aspergillosis [1].

Herbrecht et al report the radiologic findings in a cohort of 727 patients with proven or probable invasive pulmonary aspergillosis, of whom 621 had initial computed tomographic (CT) imaging available. Using the revised radiologic definitions, 1 or more wedge-shaped, lobar or segmental consolidations were present in 23.7% of patients. Moreover, nearly one-third of patients with proven invasive aspergillosis presented with a consolidation pattern but without a nodule on initial CT scan. Indeed, nonneutropenic patients were more likely to have consolidation than neutropenic patients.

We applaud the authors for producing these data so soon after the EORTC/MSGERC definitions were published and welcome the fact that their findings support the radiologic definitions proposed [1]. Determining appropriate radiologic criteria for invasive fungal disease is challenging, as the radiologic lesions are nonspecific, especially among nonneutropenic patients [4, 5]. During our deliberations, we considered other radiographic findings such as bronchial thickening with tree-in-bud lesions, ground glass opacities, micronodules, and pleural effusions as diagnostic criteria. However, the consensus was to exclude these because of their lack of specificity for pulmonary aspergillosis. Nevertheless, more data such as those presented by Herbrecht et al are clearly needed as they will be instrumental in helping reevaluate the radiologic criteria and inform future revisions of the EORTC/MSGERC definitions.

## Notes

***Potential conflicts of interest***. S. C.-A. C. reports grants from Meck Sharp & Dohme (MSD) Australia, outside the submitted work. J. P. D. reports personal fees from F2G Ltd, Gilead Sciences, and Pfizer, outside the submitted work. C. O. M. reports grants from Gilead Sciences and MSD, and advisory board fees from MSD, outside the submitted work. All other authors report no potential conflicts of interest. All authors have submitted the ICMJE Form for Disclosure of Potential Conflicts of Interest. Conflicts that the editors consider relevant to the content of the manuscript have been disclosed.
